# Role of hypoxic exosomes and the mechanisms of exosome release in the CNS under hypoxic conditions

**DOI:** 10.3389/fneur.2023.1198546

**Published:** 2023-09-15

**Authors:** Rong Yang, Zheng Li, Jing Xu, Juan Luo, Zhichuang Qu, Xin Chen, Sixun Yu, Haifeng Shu

**Affiliations:** ^1^Department of Neurosurgery, The Affiliated Hospital of Southwest Medical University, Luzhou, Sichuan Province, China; ^2^Department of Neurosurgery, Western Theater General Hospital, Chengdu, Sichuan Province, China; ^3^College of Medicine, Southwest Jiaotong University, Chengdu, Sichuan Province, China

**Keywords:** exosome, hypoxia, ischaemia, hypoxic-ischaemic injuries, neural ischaemia, neural tissue hypoxic injuries, hypoxic preconditioning, neurorehabilitation

## Abstract

Hypoxia is characterized by low oxygen levels in the body or environment, resulting in various physiological and pathological changes. The brain, which has the highest oxygen consumption of any organ, is particularly susceptible to hypoxic injury. Exposure to low-pressure hypoxic environments can cause irreversible brain damage. Hypoxia can occur in healthy individuals at high altitudes or in pathological conditions such as trauma, stroke, inflammation, and autoimmune and neurodegenerative diseases, leading to severe brain damage and impairments in cognitive, learning, and memory functions. Exosomes may play a role in the mechanisms of hypoxic injury and adaptation and are a current focus of research. Investigating changes in exosomes in the central nervous system under hypoxic conditions may aid in preventing secondary damage caused by hypoxia. This paper provides a brief overview of central nervous system injury resulting from hypoxia, and aimed to conduct a comprehensive literature review to assess the pathophysio-logical impact of exosomes on the central nervous system under hypoxic conditions.

## 1. Introduction

Molecular oxygen is essential for many biological energy and biochemical processes within cells, and many species cannot survive in anoxic environments (1, 2). Hypoxia, which is a condition in which the oxygen content in the body or environment is lower than normal, results in a series of physiological and pathological reactions (3). Although the brain accounts for only 2% of the body’s weight, it consumes 20% of the body’s oxygen, making it the organ that consumes the most energy (4). Hypoxia is a common pathological stress at all stages of central nervous system (CNS) development and can occur in various pathological conditions, including ischaemia, trauma, chronic neurodegenerative diseases, and brain tumours. Hypoxia can affect the physiology of all types of cells in complex ways, including the cell cycle, morphological structure, metabolism, proliferation, differentiation, autophagy, and apoptosis. Thus, hypoxia is a potential environmental factor associated with cell death (5). Reducing oxygen levels in the blood can be harmful to the CNS and lead to neurological diseases with significant medical and socioeconomic implications (6–9).

Human exposure to hypoxia or ischaemia in extreme environments such as high altitudes, deep diving, closed operations, or sudden illness can damage nerve cells and cause irreversible damage to CNS function. Altitude hypoxic encephalopathy is a plateau-specific disease characterized by increased intracranial pressure due to hypoxia, altitude cerebral oedema, and neuropsychiatric abnormalities. The root cause is direct hypoxic injury to brain tissue and dysfunction of brain cell energy metabolism, which reduces the function of the sodium pump on the membrane. The plateau hypoxic environment has a significant impact on the brain. High-altitude encephalopathy can be divided into acute altitude hypoxic encephalopathy and chronic hypoxic encephalopathy based on the onset time and degree of development. Additionally, the pathological effects of hypoxia on the central nervous system can be divided into multiple stages according to the length of time, such as acute hypoxia during the first 14 days, subacute hypoxia for 3–11 weeks, early chronic hypoxia for 3–6 months, and chronic hypoxia for more than 6 months (10, 11).

In recent years, increased exosome release has been detected in various experimental models of hypoxia. Exosomes are extensively engaged in the mechanisms of hypoxic injury and adaptation by providing hypoxia-specific information (12). However, the mechanisms by which hypoxia affects the secretion, composition, and function of exosomes in the CNS remain unclear.

This paper presents the mechanisms of high-altitude encephalopathy and summarizes the progress on the influence of hypoxic exosomes and the mechanisms of exosome release in the CNS under hypoxic conditions.

## 2. The impact of hypoxia on the CNS

In traumatic and nontraumatic conditions, the physiology of all types of cells is intricately affected by hypoxia at all stages of CNS development. The pathological effects of hypoxia on the CNS are acute, subacute, and chronic based on the duration of hypoxia.

Microglial cells play an important role in defending against microbial attacks and are involved in synaptic germination, neurogenesis, and brain homeostasis (13, 14). Astrocytes play an essential role in maintaining the integrity of the blood–brain barrier and the neurologic environment. These cells also play important homeostatic roles in the central nervous system through neurogenesis, neuroprotection, immune regulation, and antioxidant effects (15).

After hypoxia, the neuroplasticity and connectivity of the CNS can be easily impaired. Long-term exposure to hypoxia is associated with neuropsychiatric symptoms and an increased risk of depression (16). Demyelination, which is a vital pathological change in the CNS that can occur in depression, has been associated with the failure of oligodendrocyte progenitor proliferation and differentiation and increased oligodendrocyte apoptosis (17). However, recent research has demonstrated that inhibition of the RhoA/ROCK pathway during the early stages of hypoxia exhibits potential for enhancing oligodendrocyte progenitor cells (OPCs) proliferation and differentiation while reducing cell apoptosis (16). These findings suggest that targeting this pathway in early hypoxic conditions could augment the efficacy of antidepressants, ameliorate demyelination, and improve depressive-like behavior in chronic hypoxia. The white matter tracts of the brain, which consist of axons and myelin oligodendrocytes, can be damaged by hypoxia, resulting in neonatal cerebral palsy and delayed hypoxic leukoencephalopathy (DPHL) in adults (18).

After cerebral ischaemia, cerebral lactate concentrations increase and promote the formation of reactive astrocytes, which are a vital component of the neuroinflammatory response and functional recovery. Lactic acid has been shown to play an important anti-inflammatory role by inhibiting TNFα expression through the stabilization of NDRG2 under oxygen–glucose deprivation (OGD) conditions, which is conducive to nerve function recovery (19).

### 2.1. Characteristics and functions of exosomes

Extracellular vesicles (EVs), which are lipid bilayer-encapsulated vesicles secreted by almost all cells in living organisms, include exosomes that originate from the endosome system and have a diameter of 30-140 nm (20, 21). Early endosomes are generated through the reciprocal fusion of primary endocytic vesicles, which arise from the internalization of plasma membrane constituents. Subsequently, early endosomes mature into multivesicular bodies that harbor intraluminal vesicles. Upon fusion with the plasma membrane, these internal vesicles are released as exosomes into the extracellular milieu. Exosomes contain specific subsets of proteins from the plasma membrane, endosomes, and cytoplasm, as well as lipids, DNA, mRNA, miRNA (microRNA), and lncRNA (long noncoding RNA) (22). Exosome cargo molecules exhibit significant variability across different cell types and pathophysiological conditions.

The heterogeneity and biological function of exosomes is associated with the type of cell from which they were derived and the state of the cell at the time of exosome release. Initially, thought to remove waste from cells, exosomes have been shown to play important roles in various biological processes such as intercellular signalling, apoptosis, antigen presentation, coagulation, homeostasis, inflammation, and angiogenesis. These roles are mediated their ability to transport proteins, lipids, ribonucleic acid, and deoxypentose-nucleic acid, which influence physiological and pathological processes in a variety of diseases including neurodegenerative diseases, tumours, autoimmune diseases, and infections (23).

### 2.2. Exosomes of the central nervous system under hypoxic conditions

Under physiological and pathological conditions, exosomes can be delivered by most central nervous system (CNS) cells, such as neurons and glial cells in the brain, in a manner that relies on intracellular Ca^2+^ concentrations. These exosomes have been shown to have positive effects on neuroprotection, regeneration, development, and synaptic plasticity (24).

Currently, the purification of CNS-derived exosomes from peripheral blood has been widely used in the study of adult neurological diseases. However, their application in neonatal neurological diseases, particularly in the context of hypoxic-ischaemic encephalopathy (HIE), warrants further investigation. The cargo of CNS exosomes has the potential to serve as a biomarker for the severity of brain injury and response to therapeutic hypothermia, as well as for quantifying pharmacological responses to neuroactive therapeutic agents or adjuncts (25).

**Figure 1:**
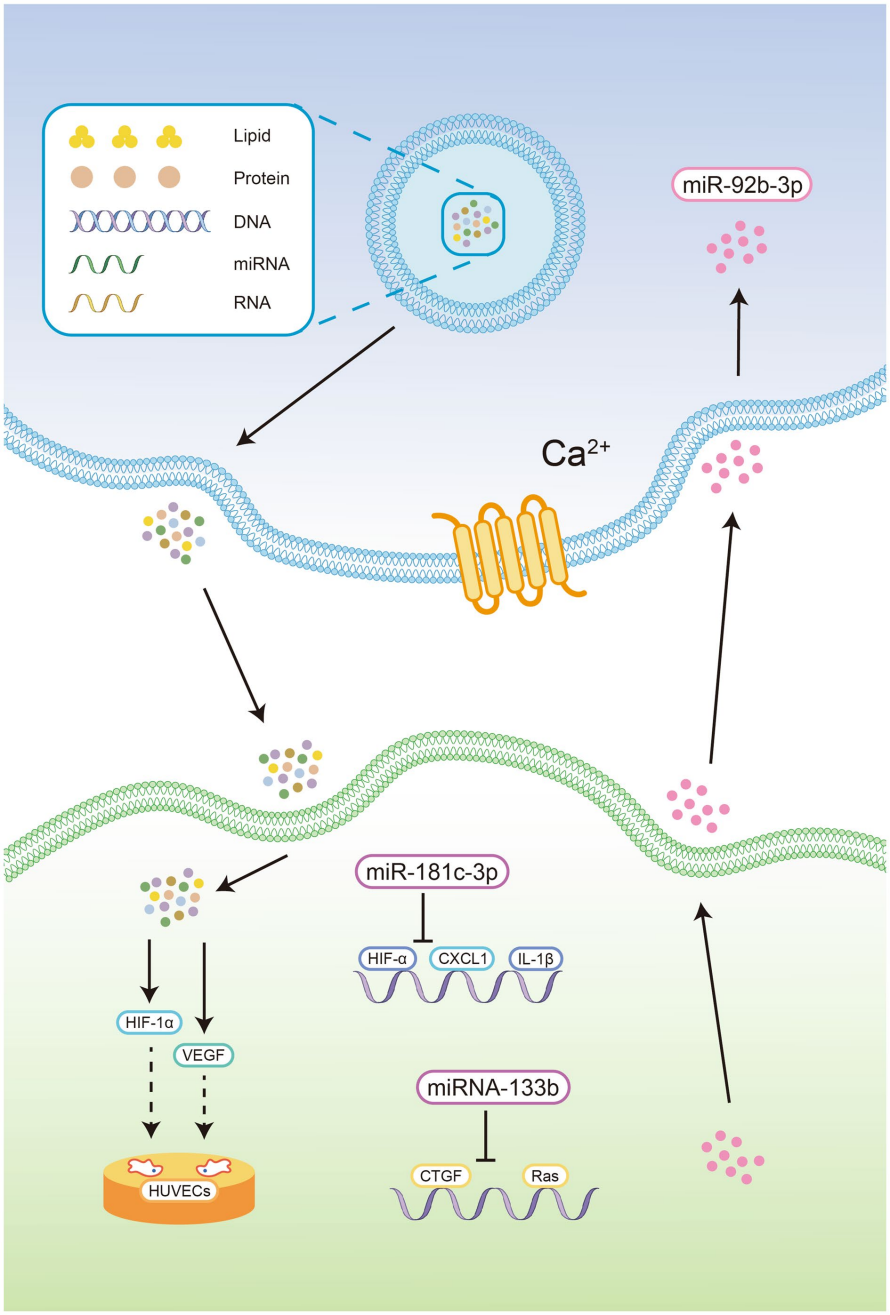
Exosomes contain proteins, lipids, DNA, mRNA, miRNA, and lncRNA. 2. Exosomes could be delivered by CNS cells in a manner that relies on intracellular Ca^2+^ concentrations. 3. Exosomes derived from OGD-treated cortical neurons reduce the expression of chemokine ligand 1 and inflammatory cytokines in astrocytes by delivering miR-181c-3p, thereby blocking the activation of inflammatory bodies in the CNS. 4. The exosome-mediated shuttling of miR-92b-3p from pretreated astrocytes to neurons. 5. MiRNA-133b in exosomes derived from cortical neurons and astrocytes inhibit the expression of growth factors and Ras homologous gene family members A. 6. Human-derived exosomes significantly increased the expression of HIF-1α and VEGF in the cerebral cortex of newborn mice, facilitating tube formation and the migration of HUVECs *in vitro* OGD model.

Exosomes mediate intercellular communication (see Figure 1) through surface interactions and intercellular miRNA shuttle mechanisms (26). Thus, exosomes not only reflect the mechanisms underlying pathological changes in the CNS but also provide a novel therapeutic agent for neuroprotection.

Oxygen-glucose deprivation (OGD) a widely accepted in vitro model of stroke that simulates apoptosis and necrosis caused by hypoxia (27). Wang et al. showed that oxygen-glucose deprivation (OGD) induced XIST expression, suppressed miR-455-3p expression and promoted TIPARP mRNA and protein expression in neurons (28). XIST affected cell proliferation and apoptosis through the miR-455-3p/TIPARP axis in OGD-induced neuronal cells. Li et al. showed that human-derived exosomes significantly increased the expression of HIF-1α and VEGF in the cerebral cortex of newborn mice, facilitating tube formation and the migration of HUVECs in an in vitro OGD model and alleviating hypoxic encephalopathy in newborn mice (29).

Exosomes derived from OGD-treated cortical neurons reduce the expression of chemokine ligand 1 and inflammatory cytokines in astrocytes by delivering miR-181c-3p, thereby blocking the activation of inflammatory bodies in the CNS (30). Furthermore, exosomes derived from OGD-treated astrocytes can be absorbed by neurons and attenuate OGD-induced neuronal death and apoptosis. This process involves the exosome-mediated shuttling of miR-92b-3p from pretreated astrocytes to neurons (31).

The investigators discovered that M1 microglia subtype actively facilitate the astrocyte-mediated deposition of chondroitin sulfate proteoglycan through the TGFβ1/SOX9 pathway, thereby impeding axonal regeneration and functional recovery. Interestingly, previous studies have demonstrated that virus-induced expression of miR133b can effectively diminish the accumulation of chondroitin sulfate proteoglycan to enhance axonal regeneration following injury (32, 33). The findings of other researchers have demonstrated that miR-379-5p possesses the ability to impede astrocyte expression, reduce CSPG expression, and suppress oxidative stress and apoptosis, ultimately promoting the recovery of motor function in rat spinal cord injury (SCI) (34).

Although reactive astrocytes tend to have detrimental effects on neighbouring cells under pathological conditions, recent research suggests that they may also play a protective role or promote brain remodelling following brain injury (35). Connective tissue growth factors and Ras homologous gene family member A can damage nerve cells under hypoxic/ischaemic conditions. However, the high expression of miRNA-133b in exosomes derived from cortical neurons and astrocytes has been shown to have a positive inhibitory effect on these factors, thereby alleviating the degree of nerve damage caused by hypoxia/ischaemia (33).

**Figure 2:**
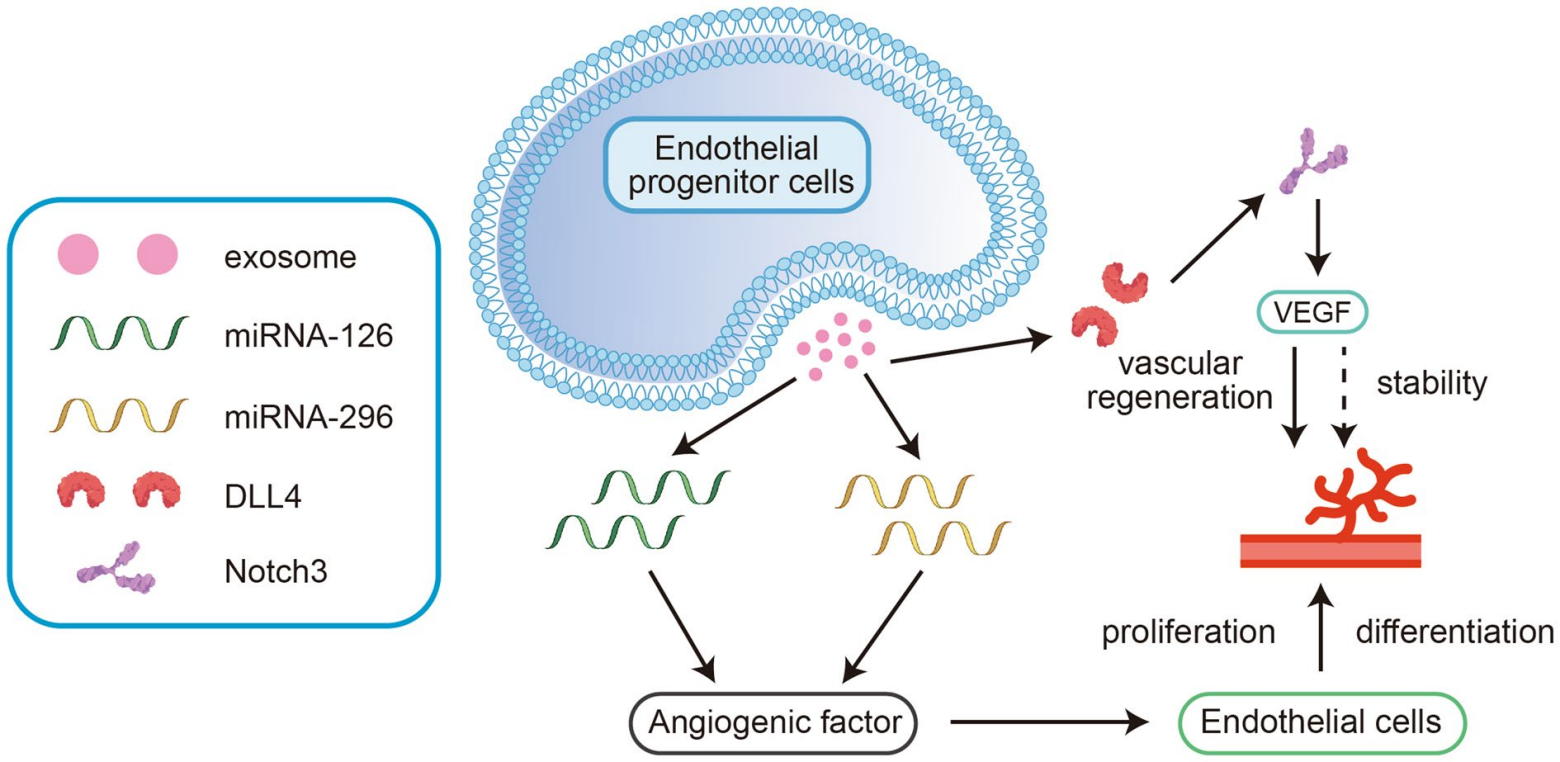
These miRNAs upregulate angiogenic factors and promote the differentiation and proliferation of endothelial cells. 2. Ligand 4 (DLL4) binds to and stimulates the Notch3 receptor. This interaction effectively maintains the stability of cerebrovascular structures and regulates vascular regeneration mediated by the vascular endothelial growth factor (VEGF) pathway.

MiRNA-126 and miRNA-296 can be detected in exosomes derived from endothelial progenitor cells. These miRNAs upregulate angiogenic factors and promote the differentiation and proliferation of endothelial cells (36). Other studies have shown that human microvascular endothelial cells and exosomes released from these cells contain abundant delta-like ligand 4 (DLL4), which binds to and stimulates the Notch3 receptor. This interaction effectively maintains the stability of cerebrovascular structures and regulates vascular regeneration mediated (see Figure 2) by the vascular endothelial growth factor (VEGF) pathway (37). Thus, exosomes promote angiogenesis by mediating the VEGF signalling pathway and maintain the stability of vascular structures to restore blood flow and oxygen supply, effectively improving nerve damage caused by hypoxia/ischaemia (See Table 1).

**Table 1:** Conceptual skeleton structure of the article’s organization approach.

Exosomes in the repair of hypoxic/ischemic injury in CNS		
(Li, 2022)	(38)	Microenvironmental factors of exosomes derived from hypoxic preconditioning human umbilical vein endothelial cells stimulate angiogenesis of MSC
(Liang, 2022)	(39)	Exosomes secreted by hypoxia-pre-conditioned adipose-derived mesenchymal stem cells reduce neuronal apoptosis in rats with spinal cord injury
(Zhang, 2022)	(40)	MiR-101a-3p mimic therapy may be a potential treatment option for spinal ischemia/reperfusion injury
(Liu, 2020)	(41)	Human neural stem cell derived extracellular vesicles have therapeutic effect on neuronal hypoxia-reperfusion injured neurons *in vitro*
(Jiang, 2018)	(42)	Exosomes protect neurons against hypoxia-reperfusion -induced injuries by suppressing miR-21-3p
(Luo, 2022)	(43)	Exosomes isolated from neural stem cells prevent cerebral injury by transferring miR-150-3p which promotes neurons proliferation by inhibiting CASP2 signaling pathway
(Huang, 2022)	(44)	EPC-derived exosomes may alleviate ischemic injury by inhibiting apoptosis and promoting angiogenesis
(Xin, 2021)	(45)	The miR-17-92 cluster enriched mesenchymal stromal cells exosomes enhanced neuro-functional recovery
(Hou, 2023)	(46)	NSC-derived exosomal miR-128-3p represents a potential therapeutic target for ischemic stroke

**Figure 3:**
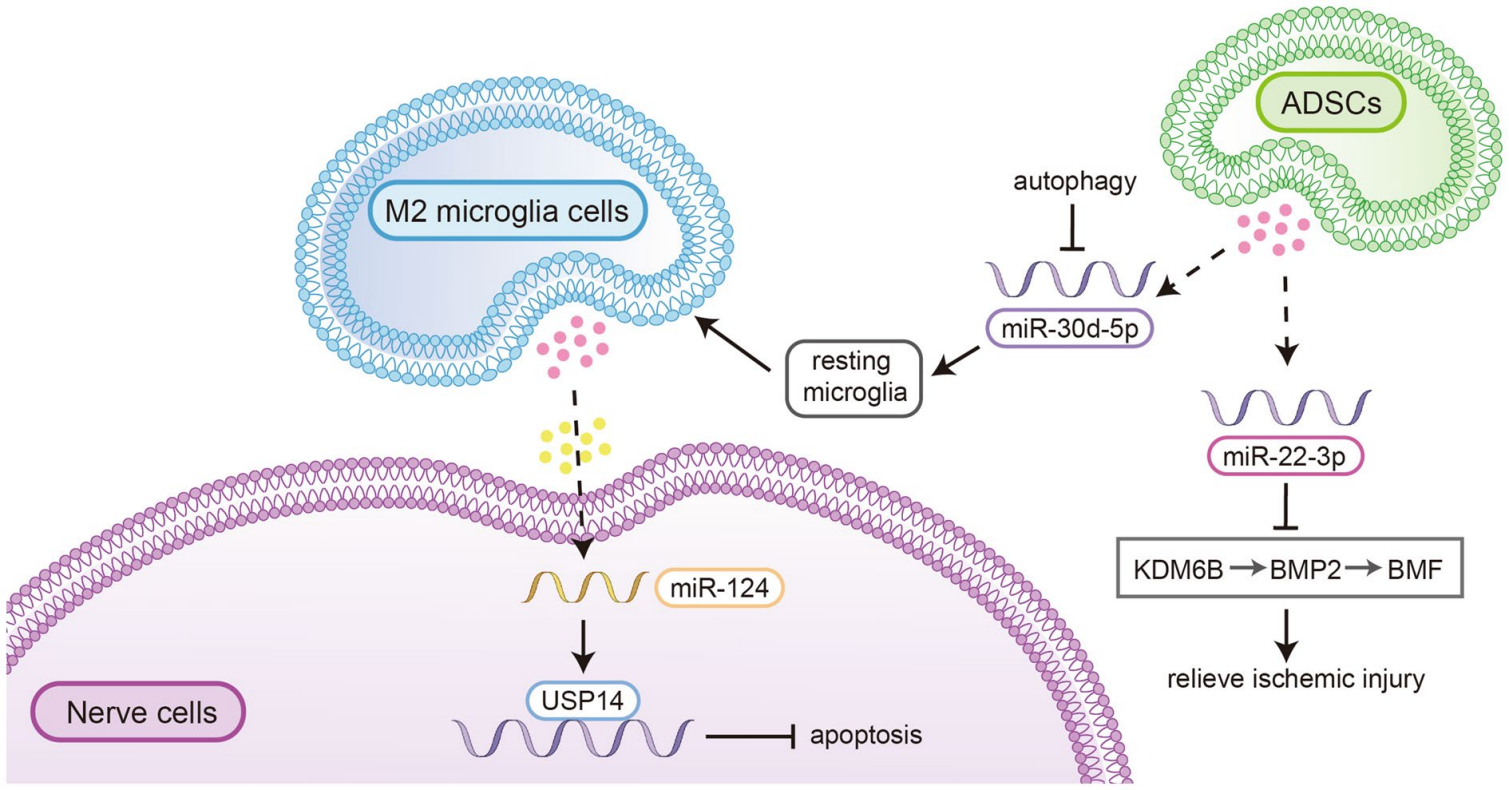
MiR-124 in exosomes derived from M2-type microglial cells could be scavenged by neurons and exert a neuroprotective effect by regulating the downstream target ubiquitin-specific peptidase 14 (USP14) to inhibit nerve cell apoptosis. 2. MiR-30d-5p in exosomes derived from ADSCs significantly reduced the area of brain infarction by inhibiting autophagy and promoting M2 microglia/macrophage polarization. 3. MiR-22-3p within extracellular vesicles derived from adipose mesenchymal stem cells could alleviate ischaemic injury by inhibiting the lysine-specific demethylase 6B/bone morphogenetic protein 2/Bcl-2 modifying factor (KDM6B/BMP2/BMF) pathway reducing the volume of cerebral infarction and promoting the recovery of neural mechanisms.

*In vitro* experiments and animal models have demonstrated that exosomes can play a vital role in neuroprotection, angiogenesis, neurogenesis, and the inhibition of inflammation and apoptosis following ischaemic stroke. For example, it has been shown that miR-124 in exosomes derived from M2-type microglial cells can be scavenged by neurons and exert a neuroprotective effect by regulating the downstream target ubiquitin-specific peptidase 14 (USP14) to inhibit nerve cell apoptosis (47). Jiang et al. reported that exosomes secreted by adipose-derived stem cells (ADSCs) play a vital role in the treatment of ischaemic injury. MiR-30d-5p in exosomes derived from ADSCs significantly reduced the area of brain infarction by inhibiting autophagy and promoting M2 microglia/macrophage polarization (48). Other researchers have demonstrated that miR-22-3p within extracellular vesicles derived from adipose mesenchymal stem cells could alleviate ischaemic injury by inhibiting the lysine-specific demethylase 6B/bone morphogenetic protein 2/Bcl-2 modifying factor (KDM6B/BMP2/BMF) pathway (see Figure 3) reducing the volume of cerebral infarction and promoting the recovery of neural mechanisms (49). Exosomes have also been shown to reduce neuronal death and improve neural deficits by regulating hypoxia-induced autophagy (50).

These findings reveal the role of miRNAs in exosomes in the treatment of ischaemic brain injury and suggest that exosomes are important intercellular mediators that protect against hypoxic/ischaemic nerve injury and postinjury repair.

### 2.3. Association of exosomes with hypoxia-inducible factor-1α

The major regulator of the cellular response to hypoxia is hypoxia-inducible factor-1 (HIF-1), which is a protein that controls the expression of more than 700 target genes involved in adaptive and pathological processes (51, 52). Genes involved in angiogenesis and energy metabolism are major targets of HIF-1. Specifically, HIF-1 regulates the expression of genes encoding erythropoietin (EPO) and vascular endothelial growth factor (VEGF), as well as genes involved in glucose transport and glycolysis, such as glucose transporter 1 (GLUT1), pyruvate dehydrogenase kinase 1 (PDK1), and lactate dehydrogenase A (LDHA) (53). HIF plays a certain role in the physiological processes of neurogenesis, nerve cell differentiation and neuronal apoptosis in the central nervous system.

Hypoxia promotes the expression and nuclear translocation of hypoxia-inducible factor 1α (HIF-1α) and hypoxia-inducible factor 2α (HIF-2α) in cells. This leads increases the protein levels of glucose transporter and epidermal growth factor receptor, which can promote plasma membrane remodelling through changes in receptor expression. Increased receptor expression can directly promote receptor activation and internalization, thereby inducing endocytosis. Ultimately, hypoxia-induced exosome release depends on HIF-1α. However, the regulation of exosome function by HIF-1α is a new area of research. The direct mechanisms by which HIF-1α regulates exosome formation, content selection, transport, and release have not yet been determined. Exosomes derived from various cell types have been shown to play a protective and therapeutic roles in different types of hypoxic diseases by reducing oxygen stress, inhibiting fibrosis, promoting angiogenesis, and inhibiting apoptosis. For example, exosomes secreted by mesenchymal stem cells overexpressing HIF-1α have been shown to enhance angiogenesis and vascular permeability under hypoxic conditions (54). Exosomes derived from umbilical cord mesenchymal stem cells have also been shown to enhance angiogenesis (55). The mammalian target of rapamycin (mTOR) signalling pathway has been confirmed to be involved in a variety of cellular processes (56), including cell growth, differentiation, development, and survival. Similarly, mTOR is a pivotal signalling pathway that protects against cerebral ischaemia–reperfusion injury (57, 58). However, Zhao et al. showed that hypoxia-induced glioma-derived exosomal miRNA-199a-3p inhibited these pathways, leading to the expansion of ischaemic injury in peritumoral neurons (59). The researchers further determined that the activation of HIF-1 α plays an important role in the mechanisms.

## 3. Exosomes in the repair of hypoxic/ischaemic injury in the CNS

Neuronal death is the primary cause of neurological deficits following spinal cord injury (SCI). Revascularization therapy is a key component of tissue engineering approaches to spinal cord repair. Mesenchymal stem cells (MSCs), which are pluripotent stem cells, have been shown to regulate diseased microenvironments by responding to microenvironmental signals. For example, the use of exosomes derived from hypoxic preconditioned human umbilical vein endothelial cells to stimulate the angiogenic potential of MSCs has been reported to be effective in promoting nerve tissue repair following spinal cord transection in rats through their proangiogenic and anti-inflammatory effects (38). These results suggest an effective angiogenic strategy for nerve tissue repair following SCI and may provide inspiration for stem cell and exosome-based therapies. Additionally, it has been found that miR-499a-5p in exosomes secreted by adipose tissue-derived stromal cells under hypoxic conditions could regulate the c-Jun N-terminal kinase 3 (JNK3)/c-Jun apoptosis signalling pathway to induce neuronal apoptosis following SCI by targeting JNK3 (39). Studies have shown that miR-101a-3p mimics can reduce apoptosis and relieve inflammation induced by spinal cord ischaemia/reperfusion injury by inhibiting the MYCN and p53 signalling pathways (40).

The brain tends to be damaged by ischaemia/hypoxia, which is closely related to stroke, hypoxic-ischaemic encephalopathy and other brain diseases. Currently, there are few effective treatments for cerebral ischaemia and hypoxia, highlighting the urgent need for the development of novel therapies. Neural stem cells are progenitor cells with the potential for division and self-renewal in the CNS (60). Some researchers have shown that extracellular vesicles derived from human neural stem cells have therapeutic effects on neuronal hypoxia reperfusion (H/R) injury *in vitro*. The mechanism mainly involves enhancing the nuclear transfer of Nrf2 and responding to oxidative stress to promote neuronal survival and inhibit neuronal apoptosis after H/R injury (41). Studies have shown that miR-21-3p plays an essential role in H/R-induced apoptosis in nerve cells, while exosomes secreted by HUVECs stimulated with H/R can alleviate H/R damage to neurons by inhibiting the expression of miR-21-3p (42). Neurons are stimulated to proliferate by miR-150-3p delivered by neural stem cell-derived exosomes (43). The main mechanism involves the suppression of the CASP2 signalling pathway by the transfer of miR-150-3p.

The results from a recent study demonstrated that exosomes derived from endothelial progenitor cells (EPCs) could alleviate ischaemic injury by promoting angiogenesis and inhibiting apoptosis in hypoxic-ischaemic tissues (44). Another study suggested that the capacity of neurofunctional recovery after stroke in the brain could be enhanced by the enrichment of miR-17-92 clusters in exosomes from pluripotent mesenchymal stromal cells. This may be ascribed to the enhancement of axonal extension and myelination, which may be mediated in part by stimulation of the phosphoinositide 3-kinase (PI3K)/Akt/mammalian target of rapamycin (mTOR) pathway induced by the downregulation of phosphatase and tensin homologue (PTEN) (45).

The latest research has shown that fibrinogen deposition inhibiting remyelination following hypoxic-ischaemic injury. However, exosomal miR-128-3p derived from neural stem cells (NSCs) has been shown to facilitate the differentiation of oligodendrocyte progenitor cells into oligodendrocytes by suppressing bone morphogenetic protein signalling, decreasing infarct volume, ameliorating neurological function following middle cerebral artery occlusion, and suggesting that miR-128-3p in NSC-derived exosomes is a potential therapeutic target for ischaemic stroke (46).

## 4. Discussion and conclusion

In this review, we have summarized the role and mechanism of exosomes in hypoxic brain injury, including fundamental knowledge on exosomes as well as relevant information regarding their involvement, diagnostic value, and therapeutic potential in cerebral hypoxic diseases. Hypoxia can modulate exosome biogenesis, cargo composition, trafficking and secretion in a context-dependent manner that is influenced by cell lineage, hypoxic severity and duration. Exosomes play vital roles in the communication between nerve cells through the proteins and miRNAs they carry. Hypoxia, which is a prominent feature of nervous system diseases, promotes exosome release and affects the composition and content of exosomes through a hypoxia-inducible factor (HIF)-dependent regulatory mechanism. Exosomes released by cells under hypoxic conditions can target cells near their parent cells or distant from their release sites, providing hypoxia-specific information and participating in the pathophysiological processes of hypoxia-related diseases. Exosomes released by neurons, neural stem cells, and astrocytes under hypoxic conditions have been shown to have significant protective effects on the nervous system, providing a new direction for nonpharmacological neuroprotection. Although hypoxia can significantly affect the release, composition, and function of exosomes, the changes in the release mechanism of exosomes under hypoxic stress and the mechanisms of targeted regulation are not yet fully understood. In this paper, we primarily explore the interplay between hypoxia and miRNA in exosomes, while the proteomics of exosomes remains incompletely understood. Furthermore, there is a lack of research on the correlation between HIF-1α and exosomes, including the direct or indirect mechanisms by which HIF-1α regulates exosome formation, content composition, transport, and release. Currently, this paper focuses on elucidating the relationship between ischemia/hypoxia and stroke-induced exosomes. We have limited understanding of the pathophysiological mechanisms underlying diseases such as high-altitude encephalopathy, chronic degenerative disorders, and tumors as well as the clinical implementation of exosomes for diagnosing and treating these ailments. Therefore, elucidating the signalling pathways related to exosome release under hypoxic conditions may contribute to the early detection and treatment of hypoxic diseases in the future and provide new insights into the pathophysiology, diagnosis, and treatment of brain hypoxic diseases.

## Author contributions

RY and HS conceived the idea of this article and supervised the research. RY performed the research, analyzed the literatures, wrote the manuscript, and participated in improving the manuscript. ZL conducted literature collection, participated in drawing the mechanism diagram of the article, and contributed to manuscript improvement. RY, ZL, JX, JL, ZQ, XC, SY, and HS reviewed the manuscript. All authors contributed to the article and approved the submitted version.

## Funding

This work was supported by Joint key project [grant numbers 2019LH01]; Department of Science and Technology of Sichuan Province [grant numbers 22CXRC0178]; Medical Innovation Project [grant numbers 21WQ040]; Hospital Management Project of Western Command General Hospital [grant numbers 2021-XZYG-B22]; Hospital Management Project of Western Command General Hospital [grant numbers 2021-XZYG-B21].

## Conflict of interest

The authors declare that the research was conducted in the absence of any commercial or financial relationships that could be construed as a potential conflict of interest.

## Publisher’s note

All claims expressed in this article are solely those of the authors and do not necessarily represent those of their affiliated organizations, or those of the publisher, the editors and the reviewers. Any product that may be evaluated in this article, or claim that may be made by its manufacturer, is not guaranteed or endorsed by the publisher.
